# Attitudes toward Management of Sickle Cell Disease and Its Complications: A National Survey of Academic Family Physicians

**DOI:** 10.1155/2015/853835

**Published:** 2015-02-22

**Authors:** Arch G. Mainous, Rebecca J. Tanner, Christopher A. Harle, Richard Baker, Navkiran K. Shokar, Mary M. Hulihan

**Affiliations:** ^1^Department of Health Services Research, Management and Policy, University of Florida, P.O. Box 100195, Gainesville, FL 32610, USA; ^2^Department of Community Health and Family Medicine, University of Florida, P.O. Box 100237, Gainesville, FL 32610-0237, USA; ^3^Department of Health Sciences, University of Leicester, 22-28 Princess Road West, Leicester LE1 6TP, UK; ^4^Department of Family and Community Medicine, Texas Tech University Health Science Center at El Paso, 9849 Kenworthy Street, El Paso, TX 79924, USA; ^5^Division of Blood Disorders, CDC, National Center on Birth Defects and Developmental Disabilities, Mail-Stop E87, 1600 Clifton Road, Atlanta, GA 30333, USA

## Abstract

*Objective*. Sickle cell disease (SCD) is a disease that requires a significant degree of medical intervention, and family physicians are one potential provider of care for patients who do not have access to specialists. The extent to which family physicians are comfortable with the treatment of and concerned about potential complications of SCD among their patients is unclear. Our purpose was to examine family physician's attitudes toward SCD management. *Methods*. Data was collected as part of the Council of Academic Family Medicine Educational Research Alliance (CERA) survey in the United States and Canada that targeted family physicians who were members of CERA-affiliated organizations. We examined attitudes regarding management of SCD. *Results*. Overall, 20.4% of respondents felt comfortable with treatment of SCD. There were significant differences in comfort level for treatment of SCD patients depending on whether or not physicians had patients who had SCD, as well as physicians who had more than 10% African American patients. Physicians also felt that clinical decision support (CDS) tools would be useful for treatment (69.4%) and avoiding complications (72.6%) in managing SCD patients. *Conclusions*. Family physicians are generally uncomfortable with managing SCD patients and recognize the utility of CDS tools in managing patients.

## 1. Introduction

Sickle cell disease (SCD) affects millions of people throughout the world and is particularly common among those whose ancestors came from sub-Saharan Africa; Spanish-speaking regions in the Western Hemisphere (South America, the Caribbean, and Central America); Saudi Arabia; India; and Mediterranean countries such as Turkey, Greece, and Italy. It is estimated that SCD affects 90,000 to 100,000 Americans, and sickle cell trait occurs among 1 in 12 African Americans [1].

Patients with sickle cell disease require comprehensive care including preventive interventions, pain management, hydroxyurea, and blood transfusions [2]. Further, complications of transfusions like iron overload are common and have significant consequences like cirrhosis, heart failure, and death [3, 4]. Due to the complex and disabling nature of sickle cell disease, appropriate ambulatory management is critical to avoid acute pain and vasoocclusive episodes and hospitalizations. One estimate has suggested that annually, United States' average hospitalization costs for SCD are $6,223 per hospitalization [5]. Interventions designed to prevent SCD complications and avoid hospitalizations are estimated to have substantial economic benefits, as the discounted lifetime cost of care averages $460,151 per patient with SCD [6].

Translation of evidence from clinical trials into health care delivery for patients with sickle cell disease needs to happen. For example, hydroxyurea, the only currently available FDA-approved medication for preventing complications of sickle cell disease, is effective. The Multicenter Study of Hydroxyurea in Patients with Sickle Cell Anemia, a multicenter landmark randomized controlled trial, clearly demonstrated that use of hydroxyurea by adult patients with sickle cell anemia resulted in a significant reduction in the frequency of pain crises, hospitalizations, and red blood cell transfusions [7]. A nine-year follow-up observational study revealed a reduction in mortality for patients taking hydroxyurea compared to study participants not taking the medication [8]. However, hydroxyurea is underused [9]. In one study at three teaching hospitals in the southeastern United States only 42% of adult SCD patients were taking hydroxyurea [10].

The extent to which family physicians are comfortable implementing such advances in treatment for SCD in clinical practice is unclear. Little information exists on current practice and use of therapies for children and adults with SCD in this setting. There are many factors that could influence a physician's attitudes toward SCD. For example, SCD is more prevalent among African Americans, and so physicians whose practices are comprised of larger proportions of African Americans might be more attuned to issues such as SCD that disproportionately affect their patient population. Physicians who have active patients with SCD may be more familiar and more comfortable with SCD patients and the disease. Physician age is another factor that may influence comfort with managing and treating SCD patients. Younger physicians may have a greater recall of details regarding less common diseases that are infrequently seen in practice. In addition, age may influence interest in use of technology in the clinical encounter. Because of the significant impact on morbidity and mortality and health care costs associated with inappropriate management of SCD [11–14], it is important to better understand current practice. A better understanding of sickle cell management and complication knowledge deficits of physicians will help to drive interventions like clinical decision support CDS systems [15, 16] and primary-specialty physician comanagement programs to improve care for the vulnerable population of people with SCD. Less common diseases such as SCD are prime candidates for CDS tools, as they can support physician knowledge and management of diseases that they do not encounter regularly in practice.

## 2. Methods

This study is an analysis of a survey conducted as part of the Council of Academic Family Medicine Educational Research Alliance (CERA). CERA is a joint initiative of all four major US academic family medicine organizations (Society of Teachers of Family Medicine (STFM), North American Primary Care Research Group (NAPCRG), Association of Departments of Family Medicine (ADFM), and Association of Family Medicine Residency Directors (AFMRD)).

The investigators submitted questions related to SCD practice and treatment for inclusion in the CERA survey. The survey was designed as an omnibus survey incorporating several distinct subprojects focusing on different topic areas. Practicing physician members of the CERA-affiliated organizations in the United States were identified for participation. Although these organizations are all headquartered in the United States, there are some members from outside the United States. This survey was limited to US based members. Since some individuals were members of multiple organizations, unique individuals were selected for the sampling frame. The study was approved by the American Academy of Family Physicians Institutional Review Board.

The survey was conducted between November, 2013, and January, 2014, and sent to 3158 physicians who are members of Council of Academic Family Medicine organizations. The potential respondents were surveyed electronically with an initial email invitation for participation. The survey was conducted through the infrastructure of STFM. The survey was introduced in an email that included a personalized greeting, a letter signed by the presidents of each of the four participating organizations urging participation, and a link to the survey. Nonrespondents were sent two follow-up emails encouraging participation. As the survey was structured as an omnibus survey, with several subprojects contained within the overall survey, it was possible for respondents to skip questions.

The survey questions for this study were developed following a review of the literature to identify key concepts and issues suggesting the need for additional knowledge. The attitudinal outcomes of interest were physicians' responses to questions related to their “comfort managing sickle cell disease patients,” “complication concerns,” “willingness to manage patients,” and “usefulness of CDS tools”.

### 2.1. Comfort Managing Patients

Comfort with overall management and pain management of SCD patients was assessed using a Likert scale (somewhat/very uncomfortable, neutral, and somewhat/very comfortable). Comfort with managing SCD patients with specific treatment options (red blood cell transfusions, hematopoietic stem cell transplant (HSCT), and hydroxyurea) was assessed as well. These options represent the main treatments available for SCD patients and represent a wide range of usage in practice, from relatively common pain management to the less frequently used HSCT.

### 2.2. Complication Concerns

Concern for SCD complications was assessed using physician's stated level of concern (somewhat/very unconcerned, neutral, and somewhat/very concerned) for known complications of SCD, including iron overload, stroke, atherosclerosis, and pneumonia.

### 2.3. Willingness to Comanage Patients with a Specialist

Willingness to comanage an SCD patient was assessed for pediatric and adult patients (somewhat/very likely, neutral, and somewhat/very unlikely).

### 2.4. Use of Clinical Decision Support Tools on SCD Care

The willingness of a physician to self-manage care of SCD patients with the assistance of a CDS tool was assessed for pediatric and adult patients (somewhat/very unlikely, neutral, and somewhat/very likely). The perceived utility of CDS tools was assessed for diagnosis of SCD, treatment of SCD, and the avoidance of complications (somewhat/very useful, neutral, and somewhat/very not useful).

### 2.5. Demographics

We collected data on age, race/ethnicity, academic rank, primary physician duty, patient time, time in clinic, proportion of patients who are African American, number of patients with SCD, and proportion of patients with SCD who are under 19 years of age from all survey participants (Table 1).

## 3. Analysis

We computed descriptive statistics to understand the general practice patterns of the survey respondents and their overall attitudes toward SCD and SCD treatment. We collapsed all of the Likert scale questions into two categories, examining the difference between those who answered the questions with a positive answer (somewhat and very comfortable, likely, and concerned) and respondents who felt neutral or responded negatively. Next, we conducted bivariate analyses with chi-square tests to compare attitudes based on respondents' proportion of African American patients (less than 10% versus 10% or greater), as well as by the presence of SCD patients in the physician's practice, and by physician age (younger than 50 versus 50 and older). We judged statistical significance at *P* ≤ 0.05.

## 4. Results

The overall number of surveys returned was 1060 for a 34% response rate. We analyzed data from the 1042 physicians who responded to at least one question on the SCD section of the survey. Table 1 shows demographic information about these physician respondents. The majority of physicians had no SCD patients, and only 15.9% had more than five SCD patients. Slightly less than half of the surveyed physicians spent 3 or more half-days in clinic. Overall, few physicians had a substantial proportion of African American patients, with only 25.7% reporting 25% or more African American patients.  Table 2 shows the bivariate analysis for comfort managing patients, complications concerns, willingness to comanage patients, and thoughts on CDS tools for the full population.  Table 3 shows differences between physicians with <10% African American patients and physicians with 10% or more African American patients.  Table 4 shows differences between physicians with no active SCD patients and those with active SCD patients. There were no significant differences between groups in relation to perceived utility of CDS for helping direct treatment or avoiding complications. A majority of all groups felt that CDS would be useful.

There were several significant differences between physicians under age 50 and those aged 50 and older. A smaller percentage of younger physicians were comfortable with overall management of SCD patients (15.7%) compared to older physicians (25.1%, *P* = 0.0002). A larger percentage of physicians who were older expressed concern for iron overload, with 66.1% expressing concern, in contrast to 55.8% of younger physicians (*P* = 0.0009). A greater percentage of younger physicians were more willing to comanage adult SCD patients (70.8%) than older physicians (64.3%, *P* = 0.03). A greater percentage of younger physicians were willing to independently manage adult SCD patients with the assistance of a CDS tool, with 38.1% of younger physicians indicating an increased likelihood, compared to 29.9% of older physicians (*P* = 0.007). A larger percentage of younger physicians saw the utility of CDS tools, with 72.5% indicating that CDS tools would be useful for the treatment of patients, compared with 66.6% of older physicians (*P* = 0.04). In addition, a greater percentage of younger physicians considered CDS tools useful for avoiding complications than older physicians (77.7% versus 67.2%, *P* = 0.0002).

## 5. Discussion

The results of this study indicate that academic family physicians have few SCD patients in their patient panel. More importantly, the results indicate that there are concerns among these primary care physicians regarding their ability to manage SCD and its complications. That said, there seems to be general agreement that a CDS tool may play a beneficial role in managing these patients especially among younger physicians.

As might be expected, more frequent interaction with SCD patients or African American patients, those at higher risk for SCD, was associated with greater comfort in managing SCD patients. Age of the physician was related to comfort managing these patients in several important ways. Older physicians appeared more comfortable with treatment and management of a complication like iron overload, potentially reflecting lifetime exposure to this patient population, while younger physicians were more likely to embrace tools that would assist them in managing patients independently.

A CDS for managing SCD received significant endorsement from this sample of academic family physicians. CDS tools have been successfully utilized in the management of care for a number of conditions [15, 16]. It appears that a CDS would have utility for both managing treatment and complications. Younger physicians were more likely to see a CDS to be particularly useful. As electronic health records become more commonplace in primary care the ability to implement a CDS for less common diseases is increased. Although there is evidence of alert fatigue with CDS for common conditions [17], the use of a CDS for SCD would not likely be perceived as an annoyance but rather as a benefit.

This study is the first study to report on family physician's comfort and attitudes with managing SCD. In addition to this strength, there are several limitations to this study. The first is that although the survey is based on a national sample of family physicians, a group that would likely encounter SCD child and adult patients, the group under study is all in academic settings. Consequently, in terms of clinical practice most academic family physicians do not practice full time. This amount of clinical practice may potentially affect their comfort with SCD. Second, even though the sample size allows us to examine responses to more than 1,000 respondents, the response rate of 34% is not exceptionally high. Thus, there may be some bias in the participants based on their comfort and interest in the questions. The low response rate may have been a result of the time of year the survey was sent out, as it was administered during the holiday season. As was clear from the practice characteristics, SCD patients are not common in the patient panels of the respondents. It is possible that individuals with no SCD patients were less likely to participate in a study on managing SCD patients. Finally, the level of training that physicians received for SCD was not assessed. Physician attitudes regarding SCD management are likely to be influenced not only by SCD patients in their care, but also by the amount of SCD-specific training they received.

In conclusion, although academic family physicians recognize issues in their comfort and ability to manage SCD patients they endorse the potential utility of CDS. Future studies could evaluate whether a CDS system could improve the quality of care and control of complications like iron overload for this vulnerable population.

## Figures and Tables

**Table 1:** Respondent demographics.

Sample size	1042
Male, %	56.6
Age, %	
Under 40	21.9
40–49	28.8
50–59	30.0
60+	19.3
Race/ethnicity, %	
White	84.2
African American	3.6
Hispanic	3.5
Asian/other	8.8
Rank, %	
Assistant professor	31.9
Associate professor	32.5
Full professor	24.6
Not applicable	11.0
Terminal degree, %	
M.D.	93.5
D.O.	5.7
Other	0.8
Primary duty, %	
Administration	26.4
Clinical teaching	51.5
Research	5.9
Faculty development	1.7
Clinical care	9.6
Nonacademic physician	0.6
Other	4.4
Patient time ≥50%, %	22.8
Time in clinic, %	
<3 half days	50.4
3–6 half days	44.6
7+ half days	5.1
% of patients who are African American, %	
<10%	46.8
10–24%	27.5
25–49%	18.4
50+%	7.3
Number of patients with SCD	
0 patients	59.6
1–4 patients	34.5
5–10 patients	14.5
11+ patients	1.4
% of SCD patients who are under 19 years of age, %	
<10%	56.8
10–24%	18.1
25–49%	14.0
50+%	11.1

**Table 2:** Physician perceptions of SCD, full sample.

	Full sample
Comfort managing patients	Comfortable
Overall management, %	20.4
RBC transfusions, %	30.8
HSCT, %	0.6
Hydroxyurea treatment, %	20.5
Pain management, %	47.8
Complication concerns	Concerned
Iron overload, %	60.9
Stroke, %	77.6
Atherosclerosis, %	45.9
Pneumonia, %	71.4
Willing to comanage patient with specialist	Likely
Pediatric patients, %	79.7
Adult patients, %	67.8
Impact of CDS on willingness to manage SCD patients	Likely
Pediatric patients, %	25.6
Adult patients, %	34.1
Perceived utility of CDS for SCD patient care	Useful
Diagnosis	22.9
Treatment	69.4
Avoiding complications	72.6

**Table 3:** Physician perceptions of SCD by percentage of patients who are African American.

	Physicians with <10% African American patients	Physicians with ≥10% African American patients	*P* value
Comfort managing patients	Comfortable	Comfortable	
Overall management, %	12.7	27.0	<0.0001
RBC transfusions, %	25.4	35.6	0.0006
HSCT, %	0.2	1.0	0.14
Hydroxyurea treatment, %	16.1	24.3	0.002
Pain management, %	42.4	52.6	0.001
Complication concerns	Concerned	Concerned	
Iron overload, %	58.6	62.8	0.18
Stroke, %	75.3	79.5	0.12
Atherosclerosis, %	43.5	48.0	0.15
Pneumonia, %	68.0	74.3	0.03
Willing to comanage patient with specialist	Likely	Likely	
Pediatric patients, %	78.2	80.9	0.31
Adult patients, %	69.8	66.0	0.20
Impact of CDS on willingness to manage SCD patients	Likely	Likely	
Pediatric patients, %	24.0	27.0	0.27
Adult patients, %	31.3	36.6	0.08
Perceived utility of CDS for SCD patient care	Useful	Useful	
Diagnosis	27.2	19.2	0.003
Treatment	68.1	70.4	0.45
Avoiding complications	70.2	74.5	0.13

**Table 4:** Physician perceptions of SCD by number of patients with SCD.

	Physicians with no SCD patients	Physicians with 1 or more SCD patients	*P* value
Comfort managing patients	Comfortable	Comfortable	
Overall management, %	9.8	36.1	<0.0001
RBC transfusions, %	21.8	45.1	<0.0001
HSCT, %	0.2	1.3	0.026
Hydroxyurea treatment, %	14.2	30.4	<0.0001
Pain management, %	39.0	61.7	<0.0001
Complication concerns	Concerned	Concerned	
Iron overload, %	58.5	64.3	0.07
Stroke, %	75.3	80.7	0.04
Atherosclerosis, %	45.4	46.7	0.70
Pneumonia, %	67.3	77.6	0.0004
Willing to comanage patient with specialist	Likely	Likely	
Pediatric patients, %	76.5	84.1	0.003
Adult patients, %	70.1	64.5	0.07
Impact of CDS on willingness to manage SCD patients	Likely	Likely	
Pediatric patients, %	23.5	28.8	0.06
Adult patients, %	30.8	38.7	0.01
Perceived utility of CDS for SCD patient care	Useful	Useful	
Diagnosis	26.9	17.0	0.0003
Treatment	68.9	70.1	0.71
Avoiding complications	72.2	73.2	0.72

## References

[1] National Heart (2009). *Disease and Conditions Index. Sickle Cell Anemia: Who Is at Risk?*.

[2] National Institutes of Health (2002). *The Management of Sickle Cell Disease*.

[3] Blinder M. A., Vekeman F., Sasane M., Trahey A., Paley C., Duh M. S. (2013). Age-related treatment patterns in sickle cell disease patients and the associated sickle cell complications and healthcare costs. *Pediatric Blood and Cancer*.

[4] Porter J., Garbowski M. (2013). Consequences and management of iron overload in sickle cell disease. *Hematology/the Education Program of the American Society of Hematology*.

[5] Steiner C. A., Miller J. L. (2006). Sickle cell disease patients in U.S. hospitals, 2004. *HCUP Statistical Brief*.

[6] Kauf T. L., Coates T. D., Huazhi L., Mody-Patel N., Hartzema A. G. (2009). The cost of health care for children and adults with sickle cell disease. *American Journal of Hematology*.

[7] Charache S., Terrin M. L., Moore R. D. (1995). Effect of hydroxyurea on the frequency of painful crises in sickle cell anemia. Investigators of the Multicenter Study of Hydroxyurea in Sickle Cell Anemia. *The New England Journal of Medicine*.

[8] Steinberg M. H., Barton F., Castro O. (2003). Effect of hydroxyurea on mortality and morbidity in adult sickle cell anemia: risks and benefits up to 9 years of treatment. *The Journal of the American Medical Association*.

[9] Lanzkron S., Haywood C., Segal J. B., Dover G. J. (2006). Hospitalization rates and costs of care of patients with sickle-cell anemia in the state of Maryland in the era of hydroxyurea. *American Journal of Hematology*.

[10] Elmariah H., Garrett M. E., de Castro L. M. (2014). Factors associated with survival in a contemporary adult sickle cell disease cohort. *The American Journal of Hematology*.

[11] Jacob E., American Pain Society (2001). Pain management in sickle cell disease. *Pain Management Nursing*.

[12] Kanter J., Kruse-Jarres R. (2013). Management of sickle cell disease from childhood through adulthood. *Blood Reviews*.

[13] Aygun B., Padmanabhan S., Paley C., Chandrasekaran V. (2002). Clinical significance of RBC alloantibodies and autoantibodies in sickle cell patients who received transfusions. *Transfusion*.

[14] Pegelow C. H., Adams R. J., McKie V. (1995). Risk of recurrent stroke in patients with sickle cell disease treated with erythrocyte transfusions. *The Journal of Pediatrics*.

[15] Player M. S., Gill J. M., Mainous A. G. (2010). An electronic medical record-based intervention to improve quality of care for gastro-esophageal reflux disease (GERD) and atypical presentations of GERD. *Quality in Primary Care*.

[16] Mainous A. G., Lambourne C. A., Nietert P. J. (2013). Impact of a clinical decision support system on antibiotic prescribing for acute respiratory infections in primary care: quasi-experimental trial. *Journal of the American Medical Informatics Association*.

[17] Kesselheim A. S., Cresswell K., Phansalkar S., Bates D. W., Sheikh A. (2011). Clinical decision support systems could be modified to reduce “alert fatigue” while still minimizing the risk of litigation. *Health Affairs*.

